# How much do adults sit? Results from the German Health Update (GEDA 2019/2020-EHIS)

**DOI:** 10.25646/10295

**Published:** 2022-09-14

**Authors:** Kristin Manz, Olga M. Domanska, Ronny Kuhnert, Susanne Krug

**Affiliations:** Robert Koch Institute, Berlin Department of Epidemiology and Health Monitoring

**Keywords:** SITTING, PHYSICAL INACTIVITY, ADULTS, HEALTH MONITORING

## Abstract

**Background:**

Sedentary behaviour is increasingly perceived as a risk factor for the development of diseases and for increased mortality. In particular, increased time spent sitting in combination with low physical activity seems to have negative health consequences.

**Methods:**

In the nationwide cross-sectional study German Health Update (GEDA 2019/2020-EHIS), the indicator ‘sitting’ was captured by the self-report of the participants.

**Results:**

For at least eight hours a day, 16.7% of women and 22.3% of men sit: Men more often than women, younger persons more often than older persons and the proportion increases significantly from the low to the high education group. Similarly, about one fifth of adults in Germany sit for at least four hours a day and do not engage in physical activity in their leisure time.

**Conclusion:**

The results indicate that preventive measures are needed to reduce time spent sitting and increase physical activity.

## Introduction

In recent years, sitting as a risk factor for the development of diseases has increasingly come into scientific and media focus. In this context, the term ‘sitting’ refers to activities performed while sitting or lying down when awake, which are associated with low energy consumption [1]. If the metabolic equivalent (MET) is used to compare energy consumption in different activities, the value for sitting behaviour is <1.5 MET (for classification: 1 MET corresponds to the energy consumption while resting and 7 METs correspond on average to the energy consumption while jogging) [2]. The negative health consequences of physical inactivity, which describes insufficient physical activity in terms of not reaching physical activity recommendations, have been known for some time [3]. Studies on the negative health effects of sitting, on the other hand, are a relatively new area of research. However, it is already clear that long periods of time spent sitting are associated with an increased likelihood of frailty and physical impairments, as well as depression and are also linked to low health-related quality of life and cognitive performance [4]. The risk of health hazards seems to increase with longer time spent sitting. In addition, it appears that the negative health effects of sitting can be at least partially compensated for by increased physical activity [5, 6]. For example, in a meta-analysis, persons who sat at least eight hours a day were 32% more likely to die from cardiovascular disease than persons who sat less than four hours a day [6]. However, this association could only be demonstrated for persons with low physical activity, while persons with high physical activity (at least 60 minutes of moderate to vigorous activity per day), which is well above current physical activity recommendations and a high amount of sitting time did not show increased mortality. Currently, there is not enough data to make specific recommendations on maximum time spent sitting per day, nor to describe the amount of physical activity needed to possibly mitigate negative health effects [3]. What is clear, however, is that increased time spent sitting in combination with very low physical activity in particular have negative health consequences. In a review article, for example, the risk of premature death is described as moderate to high, if persons sit for at least four hours a day and at the same time moderate to vigorous physical activity is less than five minutes a day [7].

**GEDA 2019/2020-EHIS**

Fifth follow-up survey of the German Health Update

**Data holder:** Robert Koch Institute

**Objectives:** Provision of reliable information on the health status, health behaviour and health care of the population living in Germany, with the possibility of European comparisons

**Study design**: Cross-sectional telephone survey

**Population:** German-speaking population aged 15 and older living in private households that can be reached via landline or mobile phone

**Sampling:** Random sample of landline and mobile telephone numbers (dual-frame method) from the ADM sampling system (Arbeitskreis Deutscher Markt- und Sozialforschungsinstitute e.V.)

**Sample size:** 23,001 respondents

**Study period:** April 2019 to September 2020

**GEDA survey waves:**

▶ GEDA 2009
▶ GEDA 2010
▶ GEDA 2012
▶ GEDA 2014/2015-EHIS
▶ GEDA 2019/2020-EHIS

Further information in German is available at www.geda-studie.de

## Indicator

The German Health Update (GEDA) is a nationwide cross-sectional survey of the resident population living in Germany. The fifth follow-up survey, GEDA 2019/2020-EHIS, took place between April 2019 and September 2020.

The indicator ‘sitting’ was captured in GEDA 2019/2020-EHIS by self-reporting by participants in a telephone survey using a fully structured questionnaire (Computer Assisted Telephone Interview, CATI). The question was taken from the Global Physical Activity Questionnaire (GPAQ) and read: ‘How much time do you spend sitting or resting in an ordinary day?’ [8]. The introduction pointed out that sitting or resting at work, at home, during transport or with friends should be considered and examples were given (sitting at a desk, sitting with friends, driving a car, bus or train, reading or watching TV). Time spent sleeping should be excluded. The response categories were: Less than four hours per day/four hours to less than six hours per day/six hours to less than eight hours per day/eight hours to less than ten hours per day/ten hours to less than twelve hours per day/twelve hours per day and more. For the analysis, a variable with four categories was formed, for which the high three response categories were combined into ‘At least eight hours per day’.

The indicator ‘sitting’ is presented stratified by gender, age and educational status (International Standard Classification of Education, ISCED [9]).

In addition, based on Dunstan et al. [7], an indicator was formed that shows persons who sit for at least four hours a day and do not engage in any moderate to vigorous physical activity in their leisure time (this can be sport, fitness or other physical activities). A more detailed description of the indicator of leisure time physical activity can be found in the GEDA Dashboard [10].

The analyses are based on data from 22,560 participants aged 18 years and older (11,863 women, 10,638 men, 59 persons with a different or no gender identity [11]) with valid information on sitting. The data of 11,775 women and 10,561 men were included for the analyses on sitting and physical activity during leisure time.

To correct for deviations of the sample from the population structure, the analyses were performed applying a weighting factor. As part of the data weighting, a design weighting was first performed for the different selection probabilities (mobile and landline network). This was followed by an adjustment to the official population figures based on age, sex, federal state, and district type (as of 31 December 2019). In addition, it is adjusted to the education distribution in the Microcensus 2017 according to the International Standard Classification of Education (ISCED classification) [12].

In this article, prevalences are reported with 95% confidence intervals (95% CI). A significant difference is assumed if the p-value calculated, taking into account the weighting and the survey design, is smaller than 0.05.

A detailed description of the GEDA 2019/2020-EHIS methodology can be found in the article German Health Update (GEDA 2019/2020-EHIS) – Background and methodology in issue 3/2021 of the Journal of Health Monitoring [13].

## Results and discussion

33.1% of women and 27.5% of men sit for less than four hours a day. Particularly high times spent sitting of at least eight hours a day were reported by 16.7% of women and 22.3% of men (Table 1). This means that men achieve this high level of time spent sitting more often than women. The higher the age, the less often women and men sit for at least eight hours a day, a clear difference can be seen between the age groups 18 to 64 years and 65 years and older. For example, while 25.4% of 18- to 29-year-old women sit for at least eight hours a day, the proportion is 7.5% for the over 65 age group (Table 1). In addition, women aged 30 to 44 years are more likely to sit less often (less than four hours a day) than women in other age groups. For men, there is no clear age effect in the group of those who sit the least.

From the low to the high education group, the proportion of adults who sit at least eight hours a day increases significantly. In particular, among 18- to 29-year-old women and men in the high education group, the proportion with a sitting time of at least 8 hours a day is high at 35.4% and 39.8% respectively (Table 1). If the differences in sitting between the education groups are stratified by age, it becomes clear that these differences do not exist among women aged 65 and older.

**Figure 1:**
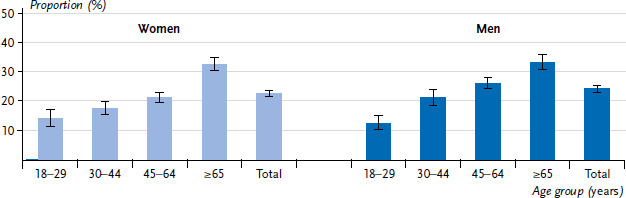
Proportion and 95% confidence intervals of women and men with at least four hours of daily time spent sitting and no leisure time physical activity by age (n=11,775 women, n=10,561 men) Source: GEDA 2019/2020-EHIS

The proportion of persons who sit for at least four hours a day and do not engage in physical activity in the leisure time is 22.6% for women and 24.3% for men (Figure 1). With increasing age, this proportion increases for both women and men. In the over 65 age group, about one-third sit for at least four hours a day and are not physically active in their leisure time.

About one fifth of adults achieve high times spent sitting, which, assuming eight hours of sleep, account for at least half of the waking time. According to current studies, this group would need to have at least 60 minutes of moderate to vigorous physical activity per day to avert negative health effects due to sitting [6, 14].

However, current data show that the majority of moderate to vigorous physical activity in the adult population does not even meet the recommended minimum of 150 minutes per week [15], so that sufficient compensation for the time spent sitting cannot be assumed. Older persons are less likely to spend high amounts of time sitting than younger persons, but older persons are also less likely to be moderately to vigorous physically active [15], so that time spent sitting of more than four hours a day are probably not compensated by this group.

Higher time spent sitting of men compared to women and a decrease of high time spent sitting with age are also reported in a current report of the Deutsche Krankenversicherung (DKV-Report 2021) [16]. Based on data from the GEDA study, it could be shown that persons in the high education group perform sedentary activities in the work context more often compared to those in the low education group [17]. Data from DKV-Report 2021 confirms a higher proportion of sitting while working among the higher educated and can at least partially explain the higher time spent sitting of this group.

Based on the reported data, it is not possible to determine which activities were performed while sitting. However, this information would be helpful for assessing the health risk due to sitting. For example, sitting while watching television is associated with an increased health risk, which can probably be attributed to unfavourable snacking behaviour during this time and relatively few interruptions in time spent sitting [5]. Moreover, depending on the activity while sitting, different preventive measures are needed to reduce or shorten the time spent sitting.

The available data on sitting was collected for the first time in the GEDA 2019/2020-EHIS study and allows the description of time spent sitting at the population level. In the interpretation, it should be taken into account that an influence on the results due to self-reporting cannot be excluded. In the context of a validation study, the validity of the question used on sitting was described as moderate [18]. It should also be noted that when the time spent sitting is presented in combination with physical activity, only leisure time physical activity was considered but not work-related physical activity or transportation from place to place. The data collection includes the time before as well as the beginning of the COVID-19 pandemic in Germany. Researchers assume that the necessary measures to control the COVID-19 pandemic have led to a significant decrease in physical activity and an increase in sitting [19]. Possible changes in sitting behaviour due to the pandemic may be reflected in this data, but due to the query of the usual time spent sitting – and the individual view of when an exceptional situation becomes a habit – cannot be differentiated for the period before and during the pandemic.

Even if there are still uncertainties as to which recommendation is suitable for the ‘optimal’ limitation of time spent sitting, it is becoming apparent that a considerable part of the adult population in Germany endangers their own health due to long times spent sitting and insufficient physical activity. Therefore, preventive measures to reduce time spent sitting are urgently needed. Multi-component interventions that include a combination of knowledge transfer and physical activity-friendly environmental design, such as offering height-adjustable desks at the workplace and reminders to take movement breaks at work, during leisure time and during transport through digital devices, are possible approaches [20, 21].

## Key statement

- Men sit at least eight hours a day more often than women (22.3% vs. 16.7%).
- 18- to 64-year-old women and men sit significantly more often for at least eight hours per day than older women and men.
- Among 18- to 64-year-old women and men in the high education group, the proportion with a sitting time of at least 8 hours a day is significantly higher than in the low education group.
- 22.6% of women and 24.3% of men sit at least four hours a day and do not engage in physical activity in their leisure time.

## Figures and Tables

**Table 1:** Time spent sitting by sex, age and education (n=11,863 women, n=10,638 men) Source: GEDA 2019/2020-EHIS

	Sitting							
	Less than four hours per day		Four to less than six hours per day		Six to less than eight hours per day		At least eight hours per day	
	%	(95% CI)	%	(95% CI)	%	(95% CI)	%	(95% CI)
Total	30.4	(29.5–31.3)	33.4	(32.4–34.3)	16.8	(16.1–17.5)	19.5	(18.7–20.3)
**Women**	**33.1**	**(31.8–34.4)**	**34.2**	**(32.9–35.5)**	**16.1**	**(15.2–17.1)**	**16.7**	**(15.7–17.7)**
**18–29 years**	23.9	(20.3–27.8)	31.5	(27.6–35.6)	19.3	(16.5–22.4)	25.4	(21.9–29.2)
Low education group	33.9	(24.2–45.2)	31.2	(21.8–42.3)	15.3	(9.6–23.5)	19.6	(12.4–29.6)
Medium education group	23.6	(19.1–28.8)	33.9	(28.8–39.5)	19.0	(15.4–23.1)	23.5	(19.1–28.6)
High education group	15.6	(10.8–21.9)	24.6	(19.2–31.0)	24.5	(18.9–31.0)	35.4	(28.7–42.6)
**30–44 years**	38.7	(35.6–42.0)	27.1	(24.4–30.0)	14.0	(12.2–16.1)	20.1	(17.9–22.6)
Low education group	57.0	(44.2–69.0)	18.8	(10.8–30.6)	9.0^*^	(3.9–19.1)	15.2^*^	(8.5–25.7)
Medium education group	40.4	(36.2–44.7)	30.7	(26.8–34.9)	11.5	(9.2–14.3)	17.4	(14.5–20.8)
High education group	27.6	(24.5–30.9)	24.3	(21.2–27.7)	20.7	(17.8–23.8)	27.4	(24.1–31.0)
**45–64 years**	33.2	(31.2–35.2)	32.4	(30.4–34.4)	16.3	(14.9–17.9)	18.1	(16.7–19.6)
Low education group	38.6	(31.4–46.3)	37.8	(30.7–45.5)	13.7	(9.3–19.9)	9.9	(6.2–15.3)
Medium education group	33.5	(31.1–35.9)	32.4	(30.1–34.8)	15.8	(14.1–17.7)	18.3	(16.5–20.3)
High education group	28.5	(26.3–30.9)	28.4	(26.3–30.7)	19.8	(18.0–21.8)	23.3	(21.3–25.4)
**≥65 years**	33.3	(31.2–35.6)	43.4	(41.0–45.8)	15.7	(14.0–17.6)	7.5	(6.2–9.1)
Low education group	33.5	(28.5–38.8)	40.6	(35.1–46.2)	17.2	(13.3–22.0)	8.8	(5.8–13.0)
Medium education group	32.9	(30.5–35.3)	44.9	(42.3–47.5)	15.2	(13.3–17.2)	7.1	(5.8–8.6)
High education group	33.7	(31.0–36.6)	45.5	(42.7–48.4)	14.4	(12.5–16.6)	6.3	(4.9–8.1)
**Men**	**27.5**	**(26.2–28.8)**	**32.6**	**(31.3–34.0)**	**17.6**	**(16.6–18.7)**	**22.3**	**(21.1–23.5)**
**18–29 years**	23.6	(20.5–27.1)	29.0	(25.6–32.6)	20.6	(17.8–23.7)	26.7	(23.7–30.0)
Low education group	22.1	(16.0–29.6)	26.9	(20.1–35.1)	28.7	(21.3–37.4)	22.3	(16.5–29.5)
Medium education group	25.3	(21.0–30.1)	32.3	(27.7–37.3)	17.3	(14.1–20.9)	25.1	(21.1–29.7)
High education group	19.5	(15.0–24.9)	20.1	(15.6–25.4)	20.6	(16.1–26.0)	39.8	(34.0–46.0)
**30–44 years**	29.8	(26.7–33.0)	26.5	(23.6–29.5)	17.0	(14.8–19.6)	26.7	(24.2–29.5)
Low education group	37.0	(25.8–49.8)	28.9	(18.9–41.5)	17.6^*^	(9.7–29.9)	16.5^*^	(9.0–28.1)
Medium education group	35.0	(30.5–39.8)	29.4	(25.2–33.9)	15.5	(12.4–19.1)	20.2	(16.9–23.9)
High education group	18.0	(15.3–21.0)	21.5	(18.7–24.5)	19.2	(16.5–22.2)	41.3	(37.7–45.0)
**45–64 years**	28.2	(26.1–30.4)	30.6	(28.5–32.8)	16.2	(14.6–17.8)	25.0	(23.1–27.1)
Low education group	35.8	(26.9–45.9)	30.9	(22.1–41.3)	9.9^*^	(5.4–17.7)	23.3	(15.5–33.5)
Medium education group	31.1	(28.1–34.2)	32.4	(29.4–35.6)	14.4	(12.2–16.8)	22.2	(19.5–25.1)
High education group	19.9	(18.0–21.9)	27.5	(25.4–29.8)	21.5	(19.6–23.5)	31.1	(28.9–33.3)
**≥65 years**	27.1	(24.8–29.5)	44.8	(42.1–47.4)	18.1	(16.2–20.3)	10.0	(8.4–11.9)
Low education group	25.8	(16.9–37.1)	34.9	(24.8–46.7)	23.5	(14.8–35.3)	15.8^*^	(8.3–28.0)
Medium education group	29.5	(26.2–33.1)	45.7	(41.9–49.5)	17.0	(14.4–20.0)	7.8	(6.0–10.0)
High education group	24.2	(22.1–26.4)	45.7	(43.3–48.2)	18.3	(16.4–20.4)	11.8	(10.2–13.5)

CI=confidence interval,

^*^ n<15
